# Reducing plastic in single-use central line insertion packs: A mixed methods observational study

**DOI:** 10.1177/0310057X251358276

**Published:** 2025-08-14

**Authors:** Alexandra R Seville, Luise Kazda, Scott McAlister, Kristen M Pickles, Katy JL Bell, on behalf of the NSW Health Net Zero Clinical Leads Program

**Affiliations:** 1 The Children’s Hospital at Westmead, Paediatric Intensive Care, Westmead, Australia; 2 NSW Health, Climate Risk & Net Zero 6079 , St Leonards, Australia; 3 University of Canberra, HEAL Global Research Centre 2234 , Canberra, Australia; 4 The University of Sydney, Wiser Healthcare Research Collaboration, Sydney, Australia; 5 The Healthcare Carbon Lab, Department of Critical Care, University of Melbourne, Melbourne, Australia

**Keywords:** Sustainable healthcare, climate change, plastic waste, intensive care, quality improvement

## Abstract

Central venous catheter (CVC) line insertion packs contain single-use plastic and metal items that are disposed of after the pack is opened, regardless of whether the item was used. This study aimed to collect data on the experiences and views of Australian clinicians who use CVC line insertion packs in paediatric critical care, elicit possible solutions to reduce waste associated with these packs, and to estimate the potential for financial and carbon footprint savings from a refined pack. This study was performed in two large paediatric tertiary referral hospitals in Sydney, Australia. Clinicians were invited to a survey and an interview to determine if and what items from a CVC line insertion pack could be excluded. Outcome measures included financial costs and embodied carbon emissions (CO_2e_). Of approximately 200 eligible clinicians who were invited, 25 (12.5%) completed the survey and 18 (9%) were interviewed (five did both). All survey respondents were willing to use a new pack that had less waste. They identified five items within the existing CVC pack as commonly non-essential. Interview data identified additional strategies for waste minimisation, including use of a trolley that allowed choice of items to use. Many clinicians expressed moral distress concerning healthcare’s impact on the environment. We calculated that a refined CVC pack without these five items would save the two participating hospitals approximately A$1400 and 230 kg CO_2e_ per year. Financial and carbon savings may be achieved through removing items that are infrequently used and/or through use of a trolley.

## Introduction

Healthcare waste is an increasing issue in hospitals across Australia and worldwide. Hospitals are the main contributors to healthcare waste, with plastics making up approximately one-third of hospitals’ waste streams. Although 40–60% is potentially able to be recycled,^1^ waste associated with surgery and procedures is frequently misclassified as clinical or hazardous waste. Australian audit studies have found that at most, only 55% of potentially recyclable materials were recycled in operating theatres,^2^ and only 49% in an intensive care unit (ICU) setting.^3^ This waste results in unnecessary costs and generation of carbon emissions from the waste disposal processes.

The introduction of single-use materials saw a widespread transition to single-use items, justified on grounds of infection control, frequent loss of reusable items, and financial savings.^4^ Pre-packaged central venous catheter (CVC) line insertion packs contain single-use plastic and metal items that are disposed of after the package has been opened, regardless of whether the item was used. Often not all of the items in the pack are used, creating unnecessary waste.^5–7^ A change back to reusable items should be the long-term goal,^8^ as the transition to renewable energy means that the carbon emissions associated with cleaning and sterilisation are substantially reduced.^9^ However, with fossil fuels still playing a dominant role in some states’ energy mix in Australia (though the transition to renewables continues apace),^10^ reusables currently may not have lower carbon emissions compared with single use.^5^

If single-use packs are to be used, then an important way to reduce their impact is to reduce the number of items included. In this project, we collected data on the experiences and views of clinicians who use paediatric CVC insertion packs. In Australia, paediatric CVCs are predominantly inserted in the settings of ICUs and perioperative anaesthetic areas of operating theatres, with some insertions also occurring in retrieval services and in neonatology units. We aimed to identify possible items that could be removed from the packs, and to elicit other possible solutions to reduce waste. We also estimated financial costs and embodied carbon emissions associated with the existing pack and potential savings associated with a revised pack.

## Methods

### Study design

This is a mixed methods study involving a cross-sectional survey and qualitative interviews of clinicians working in two paediatric tertiary care centres in Sydney, New South Wales, Australia: The Children’s Hospital at Westmead (CHW) and Sydney Children’s Hospital, Randwick (SCH).

### Participants

Participants were practising clinicians who insert CVC lines or assist in the insertion of them in critical care areas of the hospitals. These clinicians, specifically trained to insert CVC lines into large vessels, include intensivists (paediatric and neonatal), emergency medicine specialists, and anaesthetists (including those working in retrieval services). Clinicians who may be competent to carry out this procedure include doctors and some nurse practitioners. We were guided by the relevant heads of department as to what would constitute an appropriate level of training to be considered an expert in CVC insertion. For doctors working in ICU or anaesthetics, we considered consultants, Fellows and senior registrars as expert in the procedure and were included. For doctors working in paediatric emergency medicine and neonatology, we considered consultants and Fellows as expert.

### Study procedure

Eligible participants were identified by the principal investigator (AS) who was working as an ICU nurse at one of the hospitals, in collaboration with other key stakeholders: Critical Care and Surgical Directors of Clinical Operations for The Sydney Children’s Hospital Network (SCHN) and the project’s medical champion, a paediatric ICU intensivist (Supplementary material Figure 1 online). A Clinical Product Coordinator at one of the participating hospitals advised where CVC line insertion packs are predominantly used in the hospital. This assisted in identifying target departments and clinicians. Heads of department (HODs) were contacted by the Clinical Program Director or Manager. For ICU, anaesthetics, emergency and neonatology, meetings were arranged between the principal investigator and the HODs to introduce the project and a request for their departmental involvement. The HODs were asked to invite staff in their department to participate and facilitate distribution of the survey via email. HODs for CHW Interventional Radiology and for the Newborn & Paediatric Emergency Transport Service (NETS) were repeatedly contacted over a period of several months first by the Director of Clinical Operations then by the principal investigator virtually, in-person and through other department members. There was no response from Interventional Radiology and one response from NETS, who completed the survey, participated in an interview and agreed to distribute the survey to the NETS department.

Invitations for survey participation were distributed via email and QR code to all of staff within the different clinical specialties by each HOD. Inclusion criteria for the survey specified practising clinicians who insert CVCs or assist in the insertion of these lines. Selection criteria differed for the interviews, as each HOD was consulted with by the principal investigator to determine the specific staff members within the clinical specialty who had the most experience in this clinical skill. Not all survey respondents were invited for interview as some did not meet inclusion criteria as determined by the HODs.

The names of clinicians identified by their HODs as having expertise in central line insertion were provided to the principal investigator and they were invited in person or via email to be interviewed.

## Measures

### Staff views, attitudes and experiences

#### Survey

Survey data were collected using the REDCap (Research Electronic Data Capture) web-based software platform hosted at the University of Sydney.^11,12^ The survey consisted of 24 questions, operationalised as forced ranking scale, comparative scale, Likert scale and free text (Supplementary Table 1). Survey items were developed by the research team and included questions about the clinicians’ demographics, clinical experiences and views, and possible solutions to reduce waste associated with central line packs. Clinicians were asked about which items in single-use CVC line insertion packs were essential to performing the procedure and which items could potentially be removed from the pack. They were also asked about climate change and its relevance to clinical practice and patient care, using a subset of items taken from an existing measure.^13^ The survey was piloted by three clinicians who did not participate in the study. Small adjustments to the demographic questions section were made after the pilot survey. The survey was distributed via email, QR code and in person by the principal investigator.

#### Interviews

Qualitative interviews were conducted by the principal investigator, face-to-face, virtually, or by telephone, according to clinician preference. The interview guide (Supplementary Table 2) was developed by the research team with the objectives of gaining a more in-depth understanding of the clinicians’ views and experiences with the packs and eliciting their recommendations to reduce waste. Interviews were audio-recorded and transcribed verbatim to capture participant responses accurately and comprehensively. One interview pilot was conducted, and data collected was not included in the study. The interview guide itself was not altered after the pilot but the use of a visual aid depicting the single-use CVC pack in question was included for reference in future interviews.

### Financial costs

The cost of the existing single-use CVC insertion pack (price per pack, per carton and total per annum) and each individual item in the pack was obtained from the hospital’s Clinical Product Coordinator. Once we determined which items within the pack could potentially be removed according to the survey and interview data, we obtained a quote from the supplier for a new refined pack that omitted these items.

### Carbon emissions

Process-based life cycle assessment (LCA) was used to calculate embodied carbon emissions (CO_2e_). We weighed each item in the existing CVC insertion pack, and then used the International Organization for Standardization standard 14040:2000 to quantify associated kilograms of CO_2e_. Impacts, using the Intergovernmental Panel on Climate Change 2021 100-year global warming potentials, were modelled using SimaPro 9.6 (Pré, the Netherlands). Background data were obtained from the ecoinvent^14^ and AusLCI LCA^15^ databases. We used the estimates of emissions per item (kg CO_2e_) to calculate total kg CO_2e_ for the existing pack and a refined pack, and potential carbon savings.

## Data analysis

We summarised the quantitative survey data using proportions and graphical summaries, and used thematic analysis for free text responses. For the interview data, we undertook thematic analysis using a framework approach.^16^ This analysis method is an iterative process consisting of several steps including data familiarity, identifying patterns, generating codes and themes, and defining themes, using illustrative examples. Statistical analyses were conducted in SAS Studio 3.81 (SAS 9.4). Excel was used for graphical display, content analysis and thematic analysis.

## Results

Of approximately 200 staff invited to complete the survey, 25 responded and completed the survey. Of 30 clinicians invited to interview, 18 participated, with five clinicians participating in both the survey and an interview. Table 1 summarises the characteristics of the survey sample (*N* = 25). Most clinicians were aged 40 years or older, with approximately half male and half female, and half born overseas versus in Australia. Most respondents had at least one year’s experience in paediatric critical care, most commonly practising in anaesthetics or intensive care. Nearly all (*n* = 23) had a MBBS or equivalent qualification and most also held a medical specialty qualification (*n* = 20); two respondents held a nurse practitioner’s licence. Most were consultant level practitioners, followed by Fellows and then registrars. Most reported inserting between zero and 10 CVCs in the last year, while one respondent reported inserting over 50 CVCs.

**Table 1.** Survey participants (*N* = 25).

	Frequency	Percentage
Age in years		
25–40	9	36%
41–50	8	32%
51+	8	32%
Gender		
Female	12	48%
Male	13	52%
Country of birth		
Australia	11	44%
Other	14	56%
Experience in paediatric critical care in years		
<1	1	4%
1–5	7	28%
6–10	4	16%
11–15	3	12%
16+	10	40%
Clinician type		
ICU consultant	1	4%
ICU senior registrar or Fellow	6	24%
ICU nurse practitioner	2	8%
Anaesthetic consultant	10	40%
Anaesthetic senior registrar	1	4%
Neonatology consultant	2	8%
Paediatric emergency medicine consultant	2	8%
Paediatric emergency medicine Fellow	1	4%
Number of central lines inserted in past year^a^		
0–10	8	32%
11–20	8	32%
21–50	7	28%
51+	1	4%
Missing^a^	1	4%

a One participant did not answer this question.

ICU: intensive care unit.

### Survey data: staff attitudes

Table 2 summarises clinician attitudes to a possible change in the CVC pack contents. All indicated they would be very willing, or willing, to use a CVC pack that produced less waste. Most (22/25; 88%) reported they would be very comfortable, or comfortable, with using CVC packs that included fewer single-use items, with the remainder unsure. Most (19/25; 76%) indicated that they believed it was not at all likely, or not likely, that a new refined pack would affect their ability to perform the procedure or negatively impact on workflow. The majority (21/25; 84%) also reported they would be very comfortable, or comfortable, using sterilised reusable metal items within a CVC pack, and that this would not affect their ability to perform the procedure or have a negative impact on workflow (24/25, 96%).

**Table 2.** Survey participants’ attitudes to clinical change and climate change (*N* = 24^a^).

	Frequency	Percentage
Would you be willing to use a new central line pack that produces less waste?		
Very unwilling/Unwilling	0	0%
Very willing/Willing	24	100%
How comfortable would you feel in using central line insertion packs with fewer single-use items?		
Very uncomfortable/Uncomfortable	0	0%
Not sure	2	8%
Very comfortable/Comfortable	22	92%
How likely is it that a new pack with fewer single-use items would negatively impact your capacity to perform your job or workflow?		
Not at all likely/Not likely	19	79%
Somewhat likely	3	13%
Very likely/Likely	2	8%
How comfortable would you feel in using central line insertion packs with sterilised surgical metal items (needle holder, scissors and artery forceps)?		
Very uncomfortable/Uncomfortable	0	0%
Not sure	3	13%
Very comfortable/Comfortable	21	88%
How likely is it that a new pack with sterilised surgical metal items would negatively impact your capacity to perform your job or workflow?		
Not at all likely/Not likely	24	100%
Somewhat likely	0	0%
Very likely/Likely	0	0%
How much, if at all, do you think climate change is relevant to direct patient care?		
Not at all/Slightly	3	12%
Moderately	7	29%
Extremely/Very	14	58%
How much, if at all, do you think climate change is affecting the health of your patients?		
Not at all/Slightly	5	21%
Moderately	11	46%
Extremely/Very	8	34%
I feel that actions I take in my personal and/or professional life can contribute to effective action on climate change		
Strongly disagree/Disagree/Undecided	3	13%
Strongly agree/Agree	21	88%
Clinicians should have a leadership role in encouraging offices, clinics, hospitals to be as environmentally sustainable as possible.		
Strongly disagree/Disagree/Undecided	3	13%
Strongly agree/Agree	21	88%

a One participant did not respond to this set of questions on attitudes to clinical change and climate change.

Fourteen of 25 (56%) of the clinicians responded to the open response question, ‘Do you have any concerns about the current usage of single-use items in your clinical practice?’ The most common response was to highlight the excessive waste produced from single use items: ‘tremendous amount of waste’, ‘too many things that I don’t use’. One person reported concern that the provided plastic drapes are a ‘less safe way of creating a sterile field’. Another respondent commented that reducing plastic needs to be balanced against the environmental impact of energy use. One person said that they would like to reduce their waste but this can be difficult to achieve when under time pressure and bins are not available. They commented that it can be ‘more messy to keep equipment for later use and tidier to throw away’.

Table 2 also summarises clinician attitudes towards climate change and clinical care. Most clinicians (21/25; 84%) thought that climate change is moderately to extremely relevant to their patient care. Most (19/25; 76%) felt that climate change is moderately to extremely affecting the health of their patients, while 8% (2/25) felt as though this was not at all likely. The majority agreed/strongly agreed that actions they take in their personal and/or professional life can contribute to effective action on climate change (21/25; 84%) and that clinicians should have a leadership role in encouraging offices, clinics and hospitals to be as environmentally sustainable as possible (21/25; 84%).

### Survey data: items identified for possible removal from pack

**Figure 1.**
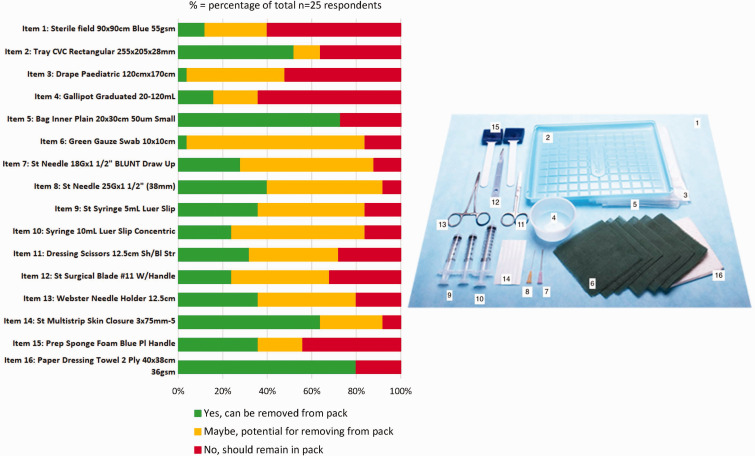
Items identified by respondents for possible removal from pack: *n* (% of total *N* = 25).

Figure 1 visually depicts single-use items included in a CVC pack. Many respondents commented that items in their packs were not exactly the same as those presented. Items identified as commonly unused by at least 50% of respondents were: paper dressing towel (20/25; 80%, Item 16), multi-strip skin closures (16/25; 64%, Item 14), bag inner plain (18/25; 73%, Item 5) and rectangular CVC tray (13/25; 52%, Item 2). Items considered essential to remain in the pack by at least 50% of respondents were: sterile field (15/25; 60%, Item 1), drape paediatric (13/25; 52%, Item 3) and gallipot (16/25; 64%, Item 4). There was no clear agreement on whether other items could be removed from the pack. We explored this further in the interviews.

### Survey data: free text responses

Fourteen of 25 clinicians responded to an open response question in the survey, ‘Do you have any suggestions about reducing usage of single use items in your clinical practice?’ Responses included: fully reusable packs, regular audit of single-use items, clinicians make their own pack as needed (‘I would rather start from scratch each time’), have fewer needles and syringes, and re-sterilisation of surgical instruments (‘it’s extremely wasteful to have single-use metal surgical equipment to an almost unethical level’, ‘it’s so heart wrenching throwing out metal instruments’). One participant commented that the whole purpose of pre-packs is to address inefficiencies and that removing items in the pack would potentially create more negative impacts such as time delays waiting for items and unnecessary risk of exposure to sterile field.

### Interview data: staff knowledge, attitudes and experiences

Eighteen medical practitioners (all at least senior registrar level) and one nurse practitioner working at the two paediatric tertiary centres were interviewed (see Supplementary Table 3 for participant demographics). The clinicians practised in intensive care medicine (*n* = 9), anaesthesia (*n* = 6), neonatology (*n* = 2) and in a neonatology retrieval service (*n* = 1). Nearly half of the sample were consultants (44%, *n* = 8), 38% (*n* = 7) were Fellows, 11% were senior registrars and one participant was a senior nurse practitioner. The mean interview duration was approximately 10 minutes.

### Awareness of generation of waste and waste disposal

Most of the clinicians perceived a significant amount of waste from single-use CVC packs, commenting that the waste is ‘noticeable’ (ID 15, intensive care nurse practitioner, 18 years’ experience, 3 CVCs/year) and something to ‘be cognisant of’ (ID 11, intensive care, 16 years’ experience, 15 CVCs/year). The disposal of single-use metal items was of particular concern: ‘it’s a significant resource … put down the drain’ (ID 3, intensive care).

Participants indicated a sense of moral distress about this waste, expressing notions of guilt, frustration, annoyance and unnecessary burden. ‘It’s not only about money, it’s about the environmental component to that’ (ID 13, NETS, 24 years’ experience); ‘I get annoyed. I think there’s a terrible amount of waste’ (ID 16, anaesthetics, 35 years’ experience); ‘It’s terrible. Single-use stuff really stresses most of us … It’s pretty in your face’ (ID 14, anaesthetics, 25 years’ experience); ‘Yeah I think it sucks’ (ID 8, intensive care, 14 years’ experience).

Clinicians commented on current suboptimal practices of waste disposal at their hospital, due to both individual clinicians’ actions and unavailability of appropriate waste disposal options (e.g. only single stream available).

‘[Waste disposal practices are] pretty bad in theatres, [often] just throwing everything in a contaminated [bin], which is a very expensive way of throwing out waste as well’ (ID 17, anaesthetics, 23 years’ experience, 21 CVCs/year).

‘People don’t throw [cardboard/paper items] into a paper recyclable bin because you’re in a hurry when you do the procedure and all just gets chucked into the same bin. So I think we’re definitely not conscious of it. And I guess the best way to reduce waste is reduce the amount of stuff you have in it, because people will just throw it away directly into the closest bin’ (ID 13, NETS, 24 years’ experience).

Multiple clinicians also reflected on organisational soft plastic recycling attempts:

‘We’ve aspired to thinking that in some way we recycle, but sadly it’s all just going to landfill at the moment’ (ID 2, anaesthetics, 26 years’ experience, 51 CVCs/year).

Even if plastic recycling processes were in place, the impact of this was questioned:

‘… it’s also not entirely clear, that recycling the soft plastics is actually being recycled. So it’s probably best not to use the stuff in the first place, not to mention the cost’ (ID 14, anaesthetics, 25 years’ experience).

Many clinicians were unsure what to do with unused items after finishing the procedure and whether these items could be reused safely without compromising infection control standards. One anaesthetist came up with their own solution, routinely taking single-use metal items (such as scissors) home to use in their personal life and to donate to other organisations because they ‘can’t stand the waste’ (ID 2, anaesthetics, 26 years’ experience, 51 CVCs/year).

### Attitudes to interventions for reducing waste from procedural packs

Clinician attitudes towards the removal of items from this pack were mostly neutral, while a few were clearly supportive: ‘would be better’ (ID 11, intensive care, 16 years’ experience, 15 CVCs/year), ‘great’ (ID 8, intensive care, 14 years’ experience) and a ‘good result’ (ID 3, intensive care). On the other hand, some perceived that reducing the contents of this pack was not warranted:

‘To be honest, what we have is not very excessive. I’ve worked in the unit where they have more stuff, but they have stuff like metal items that can be recycled’ (ID 5, intensive care, 17 years’ experience).

Another clinician highlighted the need to ensure that any changes to packs did not compromise patient safety:

‘[Reducing] plastic is important, but it should not be counterproductive to the purpose for which these packs were built, which was an emergency. Having the central line, all the things available’ (ID 18, intensive care, 24 years’ experience).

Many clinicians commented that they had previously been able to use reusable metals to perform this procedure and expressed frustration that this was no longer the case:

‘We used to reuse metals but now I’ve been told you can’t’ (ID 5, intensive care, 17 years’ experience)

and

‘… we used it for a second [time] so [not doing this] is a bit of a waste’ (ID 3, intensive care).

Some clinicians expressed concerns as to the success of any initiatives to reduce waste:

‘We have a lot of problems trying to get people to comply with any initiative. And there’s also a lot of concern about whether recycling is actually happening, so people are a little bit cynical’ (ID 14, anaesthetics, 25 years’ experience).

### Potential benefits of pack reduction

Although some clinicians saw no specific benefits from reducing items in the pack, others perceived that there could be a beneficial reduction in waste, and this could also improve staff wellbeing:

‘I think everybody in staff is quite concerned about the environment and I think that would make them feel better for doing the right thing. So I think this gets rid of mild distress with staff as well’ (ID 17, anaesthetics, 23 years’ experience, 21 CVCs/year)

‘… save on the moral injury of seeing stuff basically thrown out’ (ID 14, anaesthetics, 25 years’ experience).

Others recognised that the pack reduction could increase awareness of waste, providing an opportunity to reduce waste elsewhere:

‘… you may have some kind of knock-on effects in terms of really thinking about applying that for other procedures as well’ (ID 6, intensive care, 15 years’ experience).

‘If you can reduce the waste and I suppose it’s a good way to be very intentional about what we’re actually using while we’re using it and understanding about our resources’ (ID 6, intensive care, 15 years’ experience).

### Potential adverse impacts of pack reduction

Most clinicians did not perceive that removing common items from the single-use CVC pack would have adverse impacts, if these items were able to be easily and quickly obtained on those occasions that they were needed. This is typically the case in the two paediatric tertiary care centres where they worked:

‘Everything else is stocked in here that you need’ (ID 15, intensive care nurse practitioner, 18 years’ experience, 3 CVCs/year).

In terms of gauze, for example, one clinician commented:

‘That’s readily accessible to get more, it’s even in our bedside trolley. So if you really need it, you could grab another one’ (ID 15, intensive care nurse practitioner, 18 years’ experience, 3 CVCs/year).

Most perceived that there would be minimal impact on workflow as it was already common practice to collect additional equipment for a procedure. In addition, some commented that if there were adverse impacts these were likely to be short-lived, as practice ‘just adapts’ (ID 4, intensive care, five years’ experience) and it would just be a matter of ‘getting used to it …people are very flexible’ (ID 12, neonatology, seven years’ experience).

Most also perceived that there would be no adverse impacts on their ability to teach this procedure. One clinician believed that a reduced pack could even be beneficial:

‘I think it would make it easier to teach. The teaching would be better because you would have to think: so what do you actually need. Focus on that rather than just opening up a whole lot of stuff’ (ID 14, anaesthetics, 25 years’ experience).

However, one clinician did note possible adverse impacts on teaching during an emergency procedure, noting that it ‘would be concerning’ (ID 6, intensive care, 15 years’ experience).

### Balancing potential environmental benefits against potential risks

The need to ensure patient safety, and to balance potential environmental benefits against potential risks, was a common theme across the interviews. Clinicians commented that for more complex procedures, the use of all/additional materials might be needed to insert a CVC. In these cases, it was considered somewhat essential to have excess materials available for proceduralists ‘just in case’, given the relatively high stakes demographic of patients requiring CVC insertion. At the same time, the clinicians’ awareness of the waste produced meant that it was a balancing act between the need to maintain patient safety versus a desire to reduce waste. Clinicians acknowledged that attempting to include every item that any proceduralist might want would mean that hospitals ‘end up with a lot of unnecessary stuff’ (ID 7, anaesthetist, 37 years’ experience). Having additional materials within close proximity to the procedural space was perceived as a way of mitigating this and was thought to be ideal for workflow.

Some clinicians recognised theoretical concerns about potential contamination arising from extra movement around the sterile field collecting additional items if they were needed. However, they perceived that the actual risk of this was outweighed by potential environmental benefits of a reduced pack:

‘I think that there’s always the argument of whenever you moving things around the theatre and you’re opening up multiple packs that you increase your risk of contamination. I don’t think that that’s much of a risk and I think we’ve also got to go away from trying to go zero risk because we know that we’re balancing risk for harm to environment and waste. So I think it’s theoretical problem, but I think with care and particularly in theatres where you’ve got everything in a drawer next to you and there’s not bucket loads people in the environment it should be clean. It’s not an issue I worry about’ (ID 17, anaesthetics, 23 years’ experience, 21 CVCs/year).

### Recommendations/interventions for implementation

Finally, participants were asked whether they had any feedback or recommendations to reduce waste from single-use items packs in the clinical environment. The most common recommendation was to use a CVC procedure trolley that was stocked with all the individual items a clinician could want to perform the procedure, so they could select their preferred items. Other recommendations included tailored packs designed with end-users and providing education on sustainable practices upon orientation to the workplace (Supplementary Table 4).

### Financial costs

Table 3 presents financial costs for the current pack, and for a refined pack that omitted the five items identified as commonly not used: foam prep sponges (two pieces), gauze reduced from ten to five pieces, the number of 5 ml syringe Luer slips reduced from two to one, multi-strip skin closures, and CVC tray rectangular (substituted for another gallipot). For the refined pack a requested quote from the supplier, Multigate (Multigate Medical Products Pty Ltd, Sydney, New South Wales, Australia) (25 January 2024), indicated that a refined pack could be purchased for A$12.13. This would save A$3 per pack, and A$1434.86 per year across the two hospitals.

**Table 3.** Financial and carbon costs per CVC pack and per year for current pack and estimated savings from refined pack.

Current financial costs (Australian dollars)			Estimated financial costs after removing five items (Australian dollars)		
CVC pack unit price	Volume per year	Cost per year	Estimate for refined CVC pack (quote supplied by Multigate Medical Products Pty Ltd Australia)	Estimated costs per year	Estimated savings
A$15.13	640	A$9682.24	A$12.13	A$8247.38	A$1434.86**15% savings**
Current embodied carbon emissions (kg CO_2_e)			Estimated embodied carbon emissions after removing five items (kg CO_2e_)		
CVC pack	Volume per year	Emissions per year	Emissions for refined pack	Emissions per year	Estimated savings
1.83 kg CO_2e_	640	1171.2 kg CO_2e_	1.47 kg CO_2e_	940.8 kg CO_2e_	230.4 kg CO_2e_**20% savings**

CVC, central venous catheter.

### Carbon footprint

Table 3 presents the estimated embodied CO_2e_ for the existing pack and for the proposed refined pack that omitted contents as described above, with the impact of each item being available in Supplementary Table 5. The items with the greatest impact in the existing pack are the polyethylene drape (19% of total emissions) and the cotton gauze (18% of total emissions). The impact of the cotton gauze is notable given its mass compared with the polythene drape (13 g vs 96 g), demonstrating that natural and biodegradable items can also have significant environmental impacts. The potential reduction in CO_2e_ per pack was estimated to be 230.4 kg CO_2e_ over one year across the two hospitals.

## Discussion

To our knowledge, this is the first study to evaluate the potential waste, cost and carbon benefits of omitting items in single-use central line insertion packs (no other published reports were found on a recent scoping review conducted by our group).^4^ Clinicians working at two tertiary paediatric centres in Sydney, NSW, perceived that not all items in the current single-use CVC insertion pack were essential, and identified items for potential removal. They also suggested other solutions to waste resulting from single-use packs, including a CVC trolley where individual clinicians could select specific items they needed for the procedure, tailored packs, and educational activities about sustainable practice.

This study highlights the importance of involving front-line clinicians when designing interventions to lower healthcare’s carbon footprint. Clinicians indicated moral distress about adverse environmental impacts resulting from their everyday practice and a desire to decrease waste. Many clinicians commented on the ‘heart wrenching’ waste of metalware in particular. ‘Single-use’ and reusable metals are made of precisely the same stainless steel, but lack of metal reprocessing in the healthcare setting in Australia leads to its unnecessary disposal.^17^ Ethical issues related to this waste are further amplified by exploitation of workers, including children, by subcontractors involved in the supply chain.^18^

Some contradictory views emerged from the qualitative interview data, highlighting that whilst there was a prevalence of flexible attitudes towards adapting to change, clinicians also identified potential resistance to adopting new processes. To address this potential barrier to implementing sustainable healthcare practice, a top-down, bottom-up approach has been suggested;^19,20^ that is, combining clinician-led initiatives with endorsed local, state and federal policy^21^ so that the onus of reducing carbon emissions does not fall solely on individuals working in silos, but is mandated and standardised practice across specialties and health service delivery.

We found that even a modestly reduced pack would result in some financial and carbon savings. While the number of packs used per year at the two hospitals in this study is small, there is potential for larger savings if a reduced CVC insertion pack is adopted more widely. Further, our findings may usefully inform strategies to adopt reduced item single-use packs for other procedures done in a hospital setting. In our recent scoping review of methods to reduce waste from single-use procedure packs, eight of the 14 included studies investigated reducing the number of items in packs for: plastic, hand, head and neck, laparotomy, laparoscopic appendicectomy, paediatric tonsillectomy and other paediatric surgeries, and for vaginal births.^4^ These studies found the potential for substantial reductions in financial costs and environmental impacts, particularly CO_2e_.

Healthcare in Australia has seen widescale change from the routine use and sterilisation of reusable materials to the adoption of single-use products. Barriers to returning to reusable materials are perceived infection control standards, required workforce for sterilisation processes, and availability of renewable energy.^22^ Two studies in the scoping review evaluated potential impacts of a return to reusable central line insertion packs. McGain et al, 2012, found a higher carbon footprint for reusable CVC insertion packs in the context of coal supplied electricity in Victoria, Australia at that time.^5^ Hemberg et al, 2023, compared three packs consisting of all reusable items, reusable instruments and single-use textiles, and all single-use items. In contrast to McGain et al, they found a lower carbon footprint for the reusable pack, reflecting the low carbon electricity mix and lower energy use in their contemporary European setting.^9^ Taken together, these studies illustrate the maxim of ‘renewables make reusables better’ and serve to underscore that the long-term strategy to reduce environmental impacts from procedure packs should be to move to reusable packs.^8^ Reduced item procedure packs may be a useful interim strategy before Australia has fully transitioned to renewable energy sources.

After conducting the study, the principal investigator explored possible implementation of a new reduced item pack. The current supplier and two other suppliers (recommended by the hospital network’s clinical product coordinator) indicated that new custom packs could be created. Two companies suggested that the refined pack could include bagasse (sugar cane by-product) products with the intention of decreased environmental effect. However, bagasse has been shown to produce higher CO_2e_ than polystyrene or polypropylene alternatives.^23^ The current supplier was selected for the new refined custom CVC pack, and we anticipate rollout of this in the near future.

Our estimated savings in financial costs and carbon footprint give an indication of potential savings if a refined pack was also rolled out to other sites. The supplier for the two paediatric tertiary centres that were the focus of this study also supplies the same or similar packs to seven local health districts in New South Wales, representing 72 healthcare facilities. Whilst the financial and carbon savings at the local level were relatively small, if other sites were to also adopt a refined pack, then financial and carbon savings would be many times greater.

As this study was limited to paediatric critical care settings, the feasibility and acceptability of adopting a refined pack for adult critical care settings would need to be explored if wider rollout is planned. Considerations for state-wide procurement, as healthcare works towards decarbonisation, include requirement for carbon emissions impact to be a mandatory component in product selection criteria.^24^

Strengths of this study include its rigorous prospective design using mixed methods, and a multidisciplinary team with expertise in paediatric intensive care nursing, quantitative and qualitative methods and carbon footprinting. Our survey and interviews include representation of all important stakeholders, which might result in a greater willingness to adopt any changes that are made. Limitations include the small sample size, with low response rates amongst those invited and likely selection bias resulting in respondents who were more likely to be interested in sustainable healthcare. As the survey did not ask for respondents’ opinions on potential cost savings, we were unable to compare potential financial savings versus reduction in environmental impacts as potential motivators for change. Future research could explore the potential for environmental information to motivate policy and practice change, including the impacts of presenting data highlighting items in the packs responsible for the majority of CO_2e_.

## Conclusions

Waste from single-use CVC packs may be reduced by omitting items that are not commonly used. Future research may explore the feasibility of adoption at other clinical sites and the potential for other solutions such as a CVC trolley.

## Supplemental Material

sj-pdf-1-aic-10.1177_0310057X251358276 - Supplemental material for Reducing plastic in single-use central line insertion packs: A mixed methods observational study Supplemental material, sj-pdf-1-aic-10.1177_0310057X251358276 for Reducing plastic in single-use central line insertion packs: A mixed methods observational study by Alexandra R Seville, Luise Kazda, Scott McAlister, Kristen M Pickles and Katy JL Bell , on behalf of the NSW Health Net Zero Clinical Leads Program in Anaesthesia and Intensive Care

## Data Availability

All data are available in this paper and the Supplemental material available online.

## References

[1] NSW Circular. Plastics in healthcare: The case for circularity, https://circularaustralia.com.au/wp-content/uploads/2021/10/NSW-Circular-Plastics-in-Healthcare-Transition-Plan.pdf (2021, accessed 18 June 2025).

[2] McGain F Jarosz KM Nguyen MNHH , et al. Auditing operating room recycling: A management case report. A A Case Rep 2015; 5: 47–50. doi:10.1213/XAA.0000000000000097] 26230308 10.1213/XAA.0000000000000097

[3] Kubicki MA McGain F O’Shea CJ , et al. Auditing an intensive care unit recycling program. Crit Care Resusc 2015; 17: 135–140. doi:10.1016/S1441-2772(23)01054-2 26017132

[4] Seville ABK Pickles K Colagiuri P , et al. Reducing waste from single-use procedure packs in hospitals: A scoping review, 10.17605/OSF.IO/2MWKQ (2024, accessed 18 June 2025). PMC12967391 41688049

[5] McGain F McAlister S McGavin A , et al. A life cycle assessment of reusable and single-use central venous catheter insertion kits. Anesth Analg 2012; 114: 1073–1080. doi:10.1213/ANE.0b013e31824e9b69 22492185 10.1213/ANE.0b013e31824e9b69

[6] Qin RX Velin L Yates EF , et al. Building sustainable and resilient surgical systems: A narrative review of opportunities to integrate climate change into national surgical planning in the Western Pacific region. Lancet Reg Health West Pac 2022; 22: 100407. 35243461 10.1016/j.lanwpc.2022.100407 PMC8881731

[7] Bhangu A. Reducing the environmental impact of surgery on a global scale: Systematic review and co-prioritization with healthcare workers in 132 countries. Br J Surg 2023; 110; 7. 10.1093/bjs/znad092 PMC10364528 37079880

[8] McGain F McAlister S. Reusable versus single-use ICU equipment: What’s the environmental footprint? Intensive Care Med 2023; 49: 1523–1525. doi:10.1007/s00134-023-07256-9 37962641 10.1007/s00134-023-07256-9

[9] Hemberg L Wessberg N Leire C , et al. Environmental impact of single-use and reusable items in central venous catheter insertion kits: A life cycle assessment. Intensive Care Med 2023; 49: 662–664. doi:10.1007/s00134-023-07078-9 37166506 10.1007/s00134-023-07078-9 PMC10287769

[10] Australian Government Department of Climate Change, Energy, the Environment and Water. Australian Energy Statistics by state and territory. https://www.energy.gov.au/publications/australian-energy-statistics-state-and-territory (2024, accessed 18 June 2025).

[11] Harris PA Taylor R Minor BL , et al. The REDCap consortium: Building an international community of software platform partners. J Biomed Inform 2019; 95: 103208. doi:10.1016/j.jbi.2019.103208 31078660 10.1016/j.jbi.2019.103208 PMC7254481

[12] Harris PA Taylor R Thielke R , et al. Research electronic data capture (REDCap)—a metadata-driven methodology and workflow process for providing translational research informatics support. J Biomed Inform 2009; 42: 377–381. doi:10.1016/j.jbi.2008.08.010 18929686 10.1016/j.jbi.2008.08.010 PMC2700030

[13] Sarfaty M Bloodhart B Ewart G , et al. American Thoracic Society member survey on climate change and health. Ann Am Thorac Soc 2015; 12: 274–278. 25535822 10.1513/AnnalsATS.201410-460BC PMC5466202

[14] Wernet G Bauer C Steubing B , et al. The ecoinvent database version 3 (part I): Overview and methodology. Int J Life Cycle Assess 2016; 21: 1218–1230. doi:10.1007/s11367-016-1087-8

[15] ALCAS Australian Life Cycle Assessment Society. The Australian Life Cycle Inventory Database Initiative, www.alcas.asn.au (2016, accessed 18 June 2025).

[16] Ritchie J Lewis J Nicholls CM , et al. Qualitative research practice. London: SAGE Publications, 2003.

[17] McGain F Sussex G O’Toole J , et al. What makes metalware single-use? Anaesth Intensive Care 2011; 39: 972. 21970151

[18] Bhutta MF. Fair trade for surgical instruments. BMJ 2006; 333: 297–299. doi:10.1136/bmj.38901.619074.55 16877453 10.1136/bmj.38901.619074.55 PMC1526950

[19] Weimann L Weimann E. On the road to net zero health care systems: Governance for sustainable health care in the United Kingdom and Germany. Int J Environ Res Public Health 2022; 19: 12167. doi:10.3390/ijerph191912167 36231468 10.3390/ijerph191912167 PMC9565078

[20] Patey AM Soong C. Top-down and bottom-up approaches to low-value care. BMJ Qual Saf 2023; 32: 65–68. doi:10.1136/bmjqs-2022-014977 10.1136/bmjqs-2022-014977 36517225

[21] Australian Commission on Safety and Quality in Health Care. Joint statement on climate change and health: Working together to achieve sustainable high-quality health care in a changing climate, https://www.health.gov.au/ministers/the-hon-ged-kearney-mp/media/improving-australias-world-class-healthcare-in-a-changing-climate (2024, accessed 18 June 2025).

[22] Davies JF McGain F Sloan E , et al. A qualitative exploration of barriers, enablers, and implementation strategies to replace disposable medical devices with reusable alternatives. Lancet Planet Health 2024; 8: e937–e945. doi: 10.1016/S2542-5196(24)00241-9 39515352 10.1016/S2542-5196(24)00241-9

[23] Lightfoot SJ Grant T Boyden A , et al. Single-use synthetic plastic and natural fibre anaesthetic drug trays: A comparative life cycle assessment of environmental impacts. Br J Anaesth 2024; 133: 1465–1477. doi:10.1016/j.bja.2024.05.031 38997840 10.1016/j.bja.2024.05.031

[24] NSW Ministry of Health. NSW Health net zero roadmap 2024–2030, https://www.health.nsw.gov.au/netzero/Pages/roadmap.aspx 2025, accessed 18 June 2025).

