# Supplementary material for: Reducing plastic in single-use central line insertion packs: A mixed methods observational study
Source: Anaesth Intensive Care. 2025 Aug 14;53(6):391–401. doi: 10.1177/0310057X251358276 (PMC12619850; doi:10.1177/0310057X251358276)
Supplement: sj-pdf-1-aic-10.1177_0310057X251358276 - Supplemental material for Reducing plastic in single-use central line insertion packs: A mixed methods observational study [file sj-pdf-1-aic-10.1177_0310057X251358276.pdf]

Supplement

Supplementary Figure 1: Overview of recruitment processes

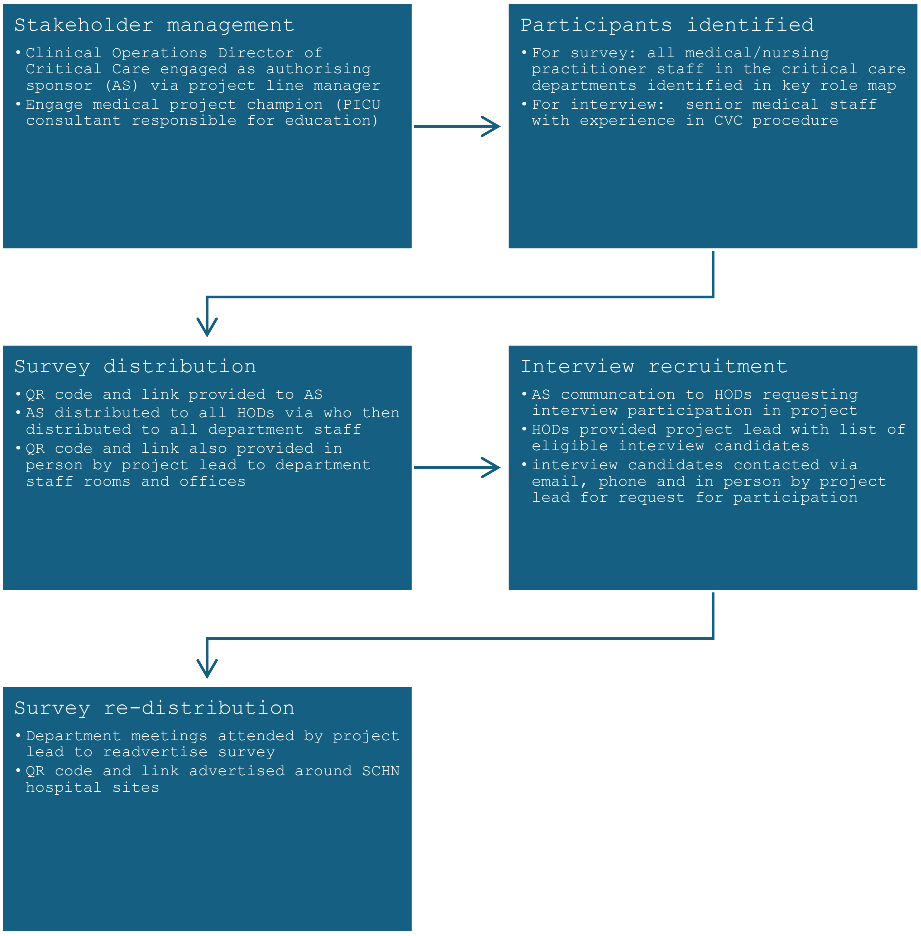

Supplementary Table 1: Staff survey

Question	Response options
1. What is your name	Free text
2. What is your email address (at work)	Free text
3. What age bracket are you in?	25–30; 31–35; 36–40; 41–45; 46–50; 51–55; 55+ years
4. How many years of experience do you have in paediatric critical care?	<1 year; 1–5 years; 6–10 years; 11–15 years; 16+ years
5. What is your gender?	Female; Male; Other/Prefer not to say
6. Were you born in Australia?	Yes/No; Country of birth
7. What is your area of work	Intensive care; neonatology; emergency medicine; anaesthetics; perioperative/operative services; nursing; interventional radiology; other – please specify your area of work
8. What is your main role at your workplace?	Free text
9. Which of the following degrees or certifications do you hold?	MBBS (or equivalent); NMBA approved Masters course for Nurse Practitioners; Medical specialty training; Other (please specify)
9.a Please indicate your specialty *(if Medical specialty training was ticked)*	Free text
9.b Please indicate which one describes you best *(if Medical specialty training was ticked)*	Resident; Registrar; Fellow; Consultant
10. How many central lines have you inserted in the last 12 months?	Free text
11. What items in the pack provided do you use every time you insert a central line?	Please tick all items that are used every time
12. What items do you think could potentially be eliminated?	Please tick all items that could be eliminated from single-use central line packs
13. Would you be willing to use a new central line pack that produces less waste?	Very unwilling – Very willing (5-point scale)
14. How comfortable would you feel in using central line insertion packs with fewer single use items?	Very uncomfortable – Very comfortable (5-point scale)
15. How likely is it that a new pack with fewer single-use items would negatively impact your capacity to perform your job or workflow?	Not at all likely – Very likely (5-point scale)
16. How comfortable would you feel in using central line insertion packs with sterilised surgical metal items (needle holder, scissors, artery forceps)?	Very uncomfortable – Very comfortable (5-point scale)

17. How likely is it that a new pack with sterilised surgical metal items would negatively impact your capacity to perform your job or workflow?	Not at all likely – Very likely (5-point scale)
18. Which of the individual items for central line insertion packs do you have easy access to on an as needed basis, if they were not included in the pack?	Please tick all that apply
19. Do you have any concerns about the current usage of single-use items in your clinical practice?	Please elaborate
20. Do you have any suggestions about reducing usage of single-use items in your clinical practice?	Please elaborate
21. How much, if at all, do you think climate change is relevant to direct patient care?	Not at all – Extremely (5-point scale)
22. How much, if at all, do you think climate change is affecting the health of your patients?	Not at all – Extremely (5-point scale)
23. I feel that actions I take in my personal and/or professional life can contribute to effective action on climate change	Strongly disagree – Strongly agree (5-point scale)
24. Clinicians should have a leadership role in encouraging offices, clinics, hospitals to be as environmentally sustainable as possible	Strongly disagree – Strongly agree (5-point scale)

Supplementary Table 2: Interview topic guide

(Note: *Questions are indicative only and exact questions reflected what was most appropriate to ask in the individual interview, adapted to interviewee responses*)

1. Can you think back to the most recent time that you used a single-use central line insertion pack.
    - Why was it needed?
    - How often do you use these packs?
    - In your opinion, what are some benefits of the current packs in terms of workflow?

2. How often are all items in the packs used in your clinical practice?
    - Describe when and why
    - Do you have any concerns about the current single use packs?

3. I’m interested in your views on unused items and potential waste associated with single-use packs,
    - Are the unused items something you reflect on?
    - What’s your understanding of how the unused items get disposed of?

4. If we were to try to reduce the contents of these packs to reduce waste, would you have any concerns about this?
    - Would you anticipate any impact on your competency to practice? (if yes, how/why)
    - Do you foresee any adverse effects? Whether this be on ability to practice, teaching, patient safety
    - Do you foresee any negative impacts on work flow?
    - What do you anticipate the main benefits to be? (i.e. of a refined pack or pack that includes sterilised reusable surgical instruments)

5. [If participant is in a managing role] What are the implications for management/ordering, ease or difficulty of change?

6. Do you have any additional thoughts/feedback about other possible solutions to reduce waste associated with these packs, or reducing usage of single-use items in your practice more generally?

Supplementary Table 3: Interview participant characteristics

ID	Clinical area	Position	Years of practice AHPRA
ID 1	CHW NICU	Fellow	16
ID 2	CHW Anaesthetics	Consultant	26
ID 3	CHW PICU	Senior Registrar	2 with AHPRA (trained overseas)
ID 4	CHW PICU	Registrar	5
ID 5	CHW PICU	Fellow	17
ID 6	CHW PICU	Fellow	15
ID 7	CHW Anaesthetics	Consultant	37
ID 8	CHW PICU	Fellow	14
ID 9	CHW Anaesthetics	Consultant	17
ID 10	CHW PICU	Fellow	9
ID 11	CHW PICU	Fellow	16
ID 12	CHW NICU	Fellow	7
ID 13	NETS	Consultant	24
ID 14	SCH Anaesthetics	Consultant	25
ID 15	CHW PICU	Nurse Practitioner	18
ID 16	SCH Anaesthetics	Consultant	35
ID 17	SCH Anaesthetics	Consultant	23
ID 18	CICU SCH	Consultant	24

CHW: The Children's Hospital at Westmead, NSW; SCH: Sydney Children's Hospital, Randwick, NSW; NETS: Newborn and Paediatric Emergency Transport Service; AHPRA: Australian Health Practitioner Regulation Agency; NICU: neonatal intensive care unit; PICU: paediatric intensive care unit; CICU: children's intensive care unit.

Supplementary Table 4: Clinician recommendations for potential solutions to reducing waste in paediatric critical care from single-use central venous catheter packs (quotes edited for brevity)

Recommendation	Reasoning (Illustrative quotes)
Procedure trolley	"I think in an ideal world you would have trolleys that have all the individual items you need in most cases and you have a very basic generic pack that has the things you always need: a gallipot, gauze, and some sort of tray like you've got there." [ID 16, anaesthetics]<br>"I think a well-stocked trolley like a central insertion trolley, which we do have, I think there's so much wastage with the packs and I guess that could be one way going forward. I think would be difficult to do one pack for everyone because I suspect PICC lines are quite different to central lines. And so I think it could be worth having a group that worked on, perhaps providing a trolley that would have all the bits and then each clinician could pick the bits that they wanted." [ID 12, neonatology]<br>"What we have here is a trolley that has everything, there, specifically for the procedure, so that you don't actually have to run anyway. If you use the top of the trolley as your surface that you set everything up on, everything is available inside it. It doesn't take a lot of extra time to open up an extra bowl or a pack or open up a scalpel. If there was items missing from the pack you'd be able to source them in seconds." [ID 16, male, anaesthetics]
New packs tailored to end-users needs	"It's just a matter of finding that sort of acceptable standard that has the minimum that everybody wants, [and] maybe a couple of things that most people want" [ID 7, anaesthetics]<br>"I guess if it's if it's done in a considered way … with the end user involved, I guess knowing where to get additional equipment that [were] in older packs .. if there's a new design." [ID 6, intensive care]
Education on sustainability in the workplace	"Recycling, reusing, sterilisation, I think there's a lot of possibilities for us to do that. It would be nice to see us engaging more in that sort of stuff." [ID 17, anaesthetics]<br>"I guess more education even as part of orientation because for instance, I've been here a few months and I didn't know that these things [single-use metals] can be recycled. So that could be beneficial as well." [ID 8, intensive care]

Supplementary Table 5: Quantity, weight, CO_{2e} for items in existing central venous catheter pack

Contribution	Quantity/pack	Weight (g/single item)	Weight (g/items in pack)	CO_{2e} (g/items in pack)
**Inflows (17)**				
Polyethylene (PE) drape	1	96	96	346
Cotton gauze	10	1.3	13	319
Dressing scissors	1	32	32	240
Sterile field	1	44	44	197
Needle holder	1	25	25	187
CVC tray	1	49	49	176
Pouch	1	6	6	102
Surgical blade with handle	1	8	8	60
Prep sponge	2	6	12	43
Gallipot	1	8	8	29
Terumo 10 ml syringe	1	8	8	28
Pouch label	1	6	6	26
Bag inner	1	28	28	22
CVC paper dressing towel	1	5	5	21
Terumo 3 ml syringe	2	7	14	21
Terumo needle 23G, 32 mm	2	0	0	4
18G drawing up needle	1	0	0	3
Total	29	-	354	1826

CVC: central venous catheter; CO_{2e}: embodied carbon emissions.
